# Development of Monoclonal Antibodies against CMP-N-Acetylneuraminate-beta-galactosamide-alpha-2,3-sialyltransferase 1 (ST3Gal-I) Recombinant Protein Expressed in* E. coli*

**DOI:** 10.1155/2015/767204

**Published:** 2015-12-10

**Authors:** Anuj Kumar Gupta, Parvinder Kaur, Harshada Patil, Pallavi Kadam, Paresh B. Bhanushali, Manoj Chugh

**Affiliations:** Yashraj Biotechnology Ltd., Plot No. C 232, TTC Industrial Area, MIDC, Navi Mumbai 400705, India

## Abstract

Aberrant glycosylation is one of the major hallmarks of cancer with altered gene expression signatures of sialyltransferases. ST3Gal-I, a sialyltransferase, is known to play a crucial role in sialylation of T antigen in bladder cancer and it has reported elevated expression in breast carcinogenesis with increased tumor progression stages. The aim of the current study is to develop new monoclonal antibodies (mAbs) against human ST3Gal-I and evaluate their diagnostic potential. We developed a repertoire of stable hybridoma cell lines producing high-affinity IgG antibodies against recombinant human ST3Gal-I, expressed in* E. coli* BL21-DE3 strain. In order to demonstrate the diagnostic value of the mAbs, various clones were employed for the immunohistochemistry analysis of ST3Gal-I expression in cancerous tissues. Antibodies generated by 7E51C83A10 clone demonstrated a strong and specific fluorescence staining in breast cancer tissue sections and did not exhibit significant background in fibroadenoma sections. In conclusion, the mAbs raised against recombinant ST3Gal-I recognize cellular ST3Gal-I and represent a promising diagnostic tool for the immunodetection of ST3Gal-I expressing cells. Specific-reactivity of clone 7E51C83A10 mAbs towards ST3Gal-I was also confirmed by immunoblotting. Therefore, our observations warrant evaluation of ST3Gal-I as a potential marker for cancer diagnosis at larger scale.

## 1. Introduction

Glycosylation is a common posttranslational modification of proteins and lipids within a cell with covalent addition of carbohydrate side chains. Altered glycosylation is very well implicated in cancer and, due to highly complex structure of sugar moieties and oligosaccharide chains, these molecules therefore give rise to large proteomic diversity. In recent years different methods have been developed to characterize and analyze them but still remain in their infancy [1, 2]. Accurate and precise addition of sugars is mediated by two enzymes critical for glycosylation known as glycosyltransferases and glycosides which are precisely and differentially expressed in various cells and tissues [3, 4].

Sialic acids are neuraminic acid residues located at terminal position of sugars in glycans and are often found linked to protein or lipid molecules. These molecules play an important role in cellular signaling during tumor formation, differentiation, and progression, which is brought about by the activity of enzymes belonging to the sialyltransferase family [5, 6].

Sialyltransferases are categorized into 4 families on the basis of the carbohydrate side chain they synthesize, namely, ST3Gal (*α* 2, 3-ST), ST6Gal (*α* 2, 6-ST), ST6GalNAc, and ST8Sia (*α* 2, 8-ST) [4]. Each sialyltransferase utilizes a specific sugar moiety as a substrate to catalyze the transfer of sialic acid to the oligosaccharide. The ST3Gal-I and ST3Gal-II utilize the type 3 oligosaccharide structure Galß1→3GalNAc-R whereas the ST3Gal-III, ST3Gal-IV, ST3Gal-V, and ST3Gal-VI use the oligosaccharide isomers Galß1→3/4GlcNAc-R [7–9].

Aberrant glycosylation is one of the major trademarks of cancer and the most common aberrant glycosylation in cancer is described in pathway of Thomsen-Friedenreich-related antigens which includes Thomsen-nouveau antigen (Tn), Sialyl-Thomsen-nouveau antigen (STn), Thomsen-Friedenreich antigen (T), and Sialyl-Thomsen-Friedenreich antigen (ST). The Tn antigen contains one residue of GalNAc alpha-O-linked to a serine/threonine residue in the polypeptide chain. Tn antigen can be sialylated to STn by ST6GalNAc-I or can be converted to core 3 structure by C3GnT. Tn antigen is converted to T antigen by T-synthase and further T antigen is converted to ST by ST3Gal-I or core-2 structure by C2GnT [10].

With the known specificities, sialyltransferase ST3Gal-I mediates the sialylation of the T antigen, a key carbohydrate tumor marker. The upregulation of ST3Gal-I has been revealed to be one of the major mechanisms responsible for the sialylation of T antigen. The T antigen is a tumor-associated structure whose sialylated form (the ST antigen) is involved in the altered expression of sialyltransferases and has been usually associated with adverse outcome and poor patient survival in cancer. Cancers of the epithelial origin such as gastric, colorectal, pancreatic, breast, and ovarian often exhibit enhanced expression of Sialyl-Tn (STn) [11, 12]. Furthermore, metastatic colorectal carcinomas show characteristic reduced expression of Tn and T tumor markers with consistent elevated expression of sialylated Tn, T, and Lewis-A and Lewis-X antigens in contrast to primary tumors. It has been widely reported that these antigens can serve as good biomarkers for cancer [13, 14].

ST3Gal-I particularly plays an important role in the sialylation of the T antigen in bladder cancer [12]. In breast carcinoma, the major carrier of T antigen is Mucin 1 (MUC1) [15, 16]. MUC-1 mucin from breast cancer cell lines (MCF-7, BT-20, and T47D) has simpler glycosylation pattern and fewer carbohydrate chains than MUC-1 from normal breast epithelial cells (MMSV1-1, MTSV1-7, and HB-2) with higher ratio of GlcN/GalN. These differences, together or alone, explain the distinct tumor specificity of some T cells and MUC-1 antibodies [17]. Solatycka et al. show that, in breast carcinoma cells, the downregulation of ST3Gal-I is directly correlated with the expression of MUC1 gene and the overexpression of MUC1 affects the carbohydrate-mediated adhesion of breast cancer cells [18].

Hence, during the present study our objective was to develop and characterize monoclonal antibodies against recombinant ST3Gal-I and hence to establish the role of enzyme and confirm its increased expression in cancerous tissues relative to normal cells. The protein was characterized using SDS-PAGE, western blot, and immunohistochemistry of breast cancer tissues. Monoclonal antibodies were generated against the purified ST3Gal-I protein and the antibodies specifically recognized proteins expressed specifically in breast cancer tissues. However, ST3Gal-I and its mAbs need to be clinically validated, at a large platform, for them to be utilized as a diagnostic tool for cancer detection and for glycan research.

## 2. Materials and Methods

### 2.1. Expression of Recombinant ST3Gal-I

Codon optimized cDNA for amino acid 1-340 of* Homo sapiens* ST3Gal-I (NCBI accession: NP_003024.1) was cloned using NcoI/HindIII sites into in-house developed pYBL01 plasmid vector containing T7 promoter system and kanamycin resistance gene for selection (data not published).* E*.* coli* strain, BL21 (DE3) (New England Biolabs Inc.), was used for recombinant protein expression. For expression, pYBL01-ST3Gal-I plasmid was transformed into BL21 (DE3) cells. The cells were cultivated at 37°C in Luria Bertani (LB) media containing kanamycin (25 *μ*g/mL) as a 10 mL culture overnight. After subculturing at *A*_600 nm_ 0.05, small scale gene expression (10 mL) was induced with a culture density *A*_600 nm_ 0.9 by addition of 1 mM of IPTG at 37°C for 3 hours. After induction, cells were harvested by centrifugation at 4°C and cell pellet was kept at −80°C for further processing.

### 2.2. Analysis of Recombinant ST3Gal-I Expression

To analyze the expression of recombinant protein (ST3Gal-I) containing N-terminal 7x His tag, 10 mL small scale culture was centrifuged at 12,000 rpm (22030 ×g) for 10 min at 4°C in Kubota centrifuge (model number 7780). Uninduced and induced cell pellet were suspended in buffer, composed of 0.125 M Tris-HCl, 10% 2 *β*-mercaptoethanol, 20% glycerol, and 0.004% bromophenol blue, pH of approximately 6.8, and analyzed by SDS-PAGE as described by Laemmli [19]. Protein bands were visualized by Coomassie blue staining.

### 2.3. Purification of Recombinant ST3Gal-I

Purification of recombinant ST3Gal-I was performed using immobilized metal affinity chromatography. A total of 9 grams wet weight of frozen cells from a 2.5 L BL21 (DE3) culture expressed at 37°C was thawed on ice and resuspended in 50 mL of lysis buffer containing 20 mM Tris, 150 mM NaCl, and 0.1% TritonX100, pH 8.0. After sonication on ice for 5 min the lysate was clarified by centrifugation at 10,000 rpm (15229 ×g) in Kubota rotor for 30 min. The pellet was washed twice with washing buffer containing 20 mM Tris, pH 8.0, 150 mM NaCl, and 0.1% Triton X-100, followed by washing twice with 20 mM Tris, pH 8.0, and 150 mM NaCl. Final pellet (inclusion bodies) was dissolved in 60 mL of equilibration buffer containing 8 M urea, 20 mM Tris, 300 mM NaCl, and 1 mM DTT, pH 8.0. The dissolved sample was filtered through a 0.2 *μ*m pore size filter apparatus (Nalgene, Rochester, NY). A Single 20 mL Chelating Sepharose (GE Healthcare, India) IMAC column, charged with 0.1 M nickel sulfate, was preequilibrated with 20 mM Tris-Cl, pH 8.0, 8 M urea, 300 mM NaCl, and 1 mM DTT (equilibration buffer) prior to loading the filtered sample. Flow rate was set at 3 mL/min throughout the run. Protein flow through was collected as the sample passed through the columns. After loading was completed, columns were washed with equilibration buffer for at least ten column volumes (CV) until absorbance at 280 nm became stable at baseline. Washing was done with 5 CV of equilibration buffer (20 mM Tris-Cl, pH 8.0, 8 M urea, 300 mM NaCl, and 1 mM DTT) containing 50 mM imidazole. For the elution of protein, 5 CV of elution buffer (20 mM Tris-Cl, pH 8.0, 8 M urea, 300 mM NaCl, 1 mM DTT, and 300 mM imidazole) was applied. Protein fractions were collected at 20 mL each and analyzed by 12% SDS-PAGE. Recombinant ST3Gal-I containing fractions were pooled with final addition of 0.2 M arginine and protein was concentrated in PEG 20000 for 16 hours. Concentrated sample was dialyzed against 20 mM Tris-Cl, pH 5.0, 0.2 M arginine, and 50% glycerol at room temperature (25°C) for 16 hours. The dialyzed sample was filtered through a 0.2 *μ*m pore size filter apparatus (Nalgene, Rochester, NY). Total protein concentration was estimated spectrophotometrically at *A*_280 nm_.

### 2.4. Generation of Monoclonal Antibodies

Five cohorts of three 6–8-week-old female BALB/c mice each were immunized by a subcutaneous injection of 10–50 *μ*g of recombinant ST3Gal-I using hybridoma technology as described by Kohler and Milstein [20]. For an initial immunization, the antigen was emulsified in Freund's complete adjuvant (Sigma). Subsequent immunizations at days 21, 42, and 63 were performed with the antigen emulsified in Freund's incomplete adjuvant. Antisera was collected two weeks after each injection and tested for the presence of ST3Gal-I-specific antibodies by indirect ELISA. The mouse with the highest antibody titer was boosted intraperitoneally, twice (24 hours interval between two boosters) with 15 *μ*g of ST3Gal-I dissolved in PBS, 2 days before the cell fusion. Mouse splenocytes were fused with Sp2/0-Ag 14 mouse myeloma cells using Hybri-Max polyethylene glycol solution (50% W/v) (Sigma-Aldrich-P7181). Hybrid cells were selected in growth medium supplemented with HAT (hypoxanthine, aminopterin, and thymidine) (50x HAT media supplement, Sigma-Aldrich-H0262). Culture supernatants of hybridoma clones were screened by ELISA against recombinant ST3Gal-I protein and the positive clones were subcloned by limiting dilution. Subcloning was performed twice to ensure monoclonal behavior and stability of the clones. Hybridoma cells were grown in complete Dulbecco's modified Eagle's medium (DMEM, SIGMA) supplemented with 15% fetal bovine serum (FBS, Gibco), Glutamax (Gibco), and antibiotics antibacterial antimycotic (Gibco). The resulting hybridomas were screened for the presence ST3Gal-I protein specific antibodies by indirect antibody capture ELISA using Fc specific HRP conjugated anti-mouse IgG to select clones secreting immunoglobulin's of only IgG isotype. Clones secreting antibodies reactive to His-tagged recombinant protein were neglected. The isotype of generated mAbs was determined with a mouse monoclonal iso-typing kit (SIGMA) according to the manufacturer's instructions.

### 2.5. Immunoblotting

Recombinant ST3Gal-I purified antigen was resolved by sodium dodecyl sulphate polyacrylamide gel electrophoresis (SDS-PAGE) and transferred on nitrocellulose membranes. Blots were probed with 10 mL of monoclonal antibodies (100 ng/mL) purified from ascitic fluid raised* in vivo* from* Balb/c* mice. Bound mAbs were detected with the Fc specific HRP-conjugated goat anti-mouse immunoglobulin of IgG isotype (Sigma), followed by a calorimetric reaction with TMB substrate (Sigma). Protein loading and transfer efficiency were monitored by Coomassie blue [21] and Ponceau S staining, respectively, as described elsewhere.

### 2.6. Immunohistochemistry

Formalin fixed paraffin embedded blocks of breast cancer tissue and fibroid adenoma were received from Pathology Department, All India Institute of Medical Sciences, New Delhi. The 4 *μ*M thick sections were treated by Citra in steamer for 30 min. Sections were blocked in 1xTBS/10% normal goat serum for 20 min at RT. Sections were incubated with primary antibody diluted 1 : 50 in 1xTBS overnight at 4°C. Sections were rinsed three times in TBS for 5 min each at RT and were incubated with secondary antibody diluted 1 : 1000 in 1xTBS for 60 min at RT in dark. Sections were then rinsed three times in TBS for 5 min each at RT. Coverslips were mounted on slides with DAPI (Invitrogen, Prolong Gold antifade reagent with DAPI). The sections were imaged with the Olympus Phase contrast inverted microscope, CKX 41SF.

## 3. Results 

### 3.1. Analysis of Recombinant ST3Gal-I

**Figure 1:**
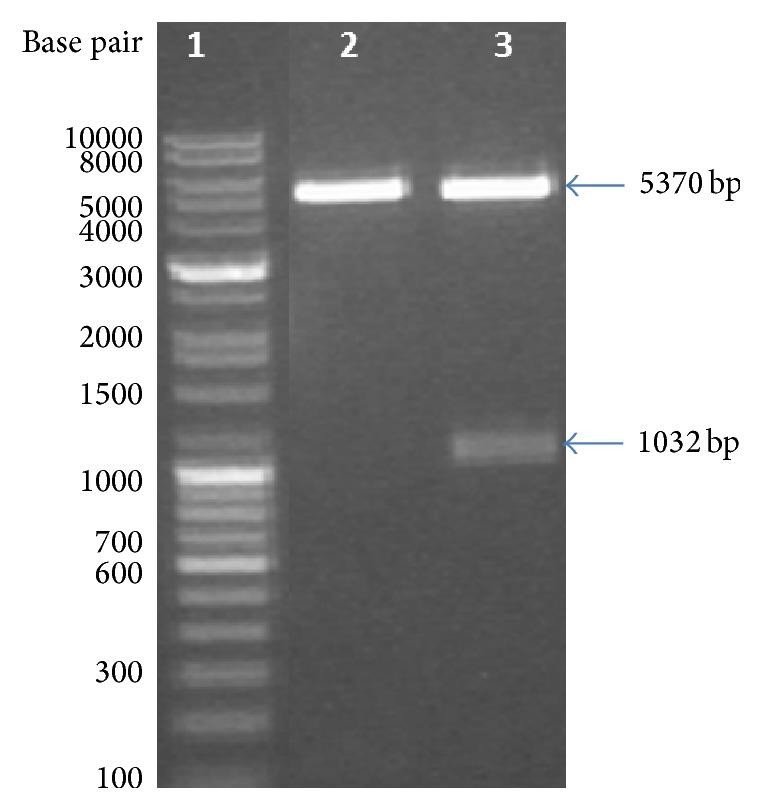
Characterization of pYBL01-ST3Gal-I clone: lane 1: 1 Kb DNA ladder, Merck; lane 2: Backbone vector digested with NcoI/HindIII enzymes and the expected band size is 52 bp and 5370 bp; lane 3: pYBL01-ST3Gal-I clone, digested with NcoI/HindIII enzymes and the expected band sizes are 1032 bp and 5370 bp.

**Figure 2:**
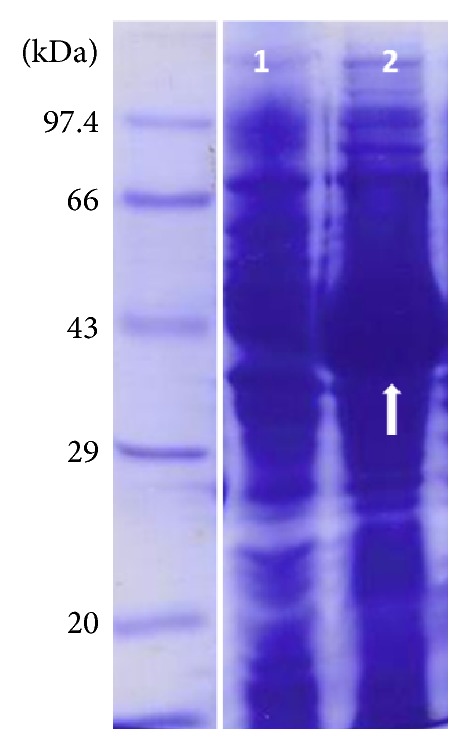
Coomassie blue stained SDS-PAGE analysis of recombinant ST3Gal-I in* E*.* coli* strain BL21 (DE3): lane 1: uninduced rST3Gal-I culture; lane 2: induced rST3Gal-I culture. Protein molecular weight marker from Merck was used. Arrow showed induced recombinant protein.

The DNA construct of ST3Gal-I (pYBL01-ST3Gal-I) was characterized with NcoI/HindIII enzyme (Figure 1). The expression of ST3Gal-I proteins was performed using* E. coli* strain, BL21 (DE3), at induction temperatures 37°C with 1 mM IPTG concentrations for 3 hours. As observed in the localization study, protein is expressed as inclusion bodies in its insoluble form. After induction with IPTG at 37°C, the expressed protein products of induced and uninduced recombinant bacterial containing the Recombinant ST3Gal-I were compared (Figure 2).

### 3.2. Purification of rST3Gal-I

**Figure 3:**
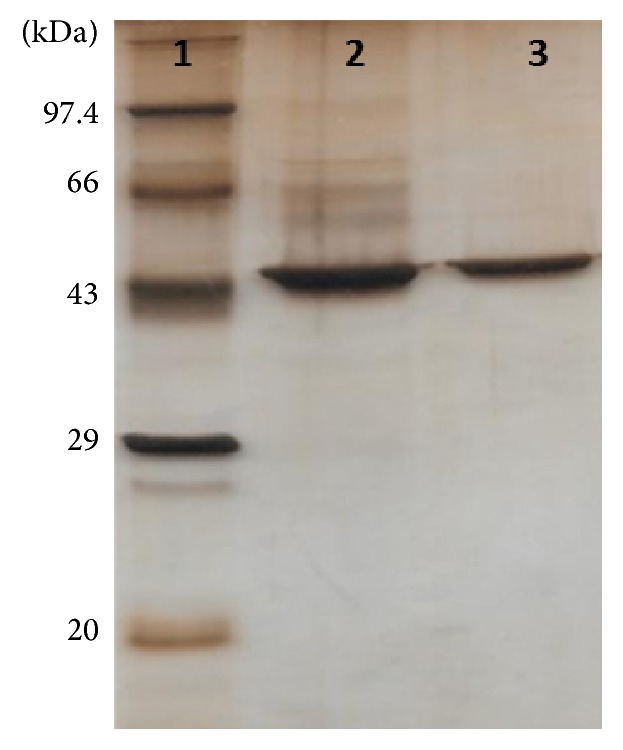
Silver stained SDS-PAGE analysis of purified recombinant rST3Gal-I. Lane 1: protein Molecular weight marker from Merck; lanes 2 and 3: purified recombinant ST3Gal-I protein (2 *μ*g and 1 *μ*g, resp.).

Recombinant ST3Gal-I purification was performed on the basis of its nickel binding affinity using Chelating Sepharose IMAC column with elution at 300 mM imidazole. The protein was concentrated within PEG 20000 and finally dialyzed into the buffer with 50% glycerol as stabilizing agent. A band of purified protein corresponding to approximately 44 kDa was observed, as illustrated by reducing 12% SDS-PAGE, silver stained (Figure 3). We got the purity of desired rST3Gal-I which is greater than 95% on the bases of SDS-PAGE profile. The yield of purified rST3Gal-I was found to be approximately 14 mg per 2.5 L of bacterial culture.

### 3.3. Monoclonal Antibody Development

An array of monoclonal antibodies was generated against the injected rST3Gal-I (with only nonrepeated sequences) and subcloned twice by limiting dilution method. We could identify 7 monoclonal antibodies against recombinant protein, which did not recognize His tag and normal human serum as described elsewhere. 7E5 1C8 3A10 monoclonal antibodies exhibited strong reactivity to ST3Gal-I., for ELISA, western blot, and immunofluorescence studies.

### 3.4. Western Blot Analysis

**Figure 4:**
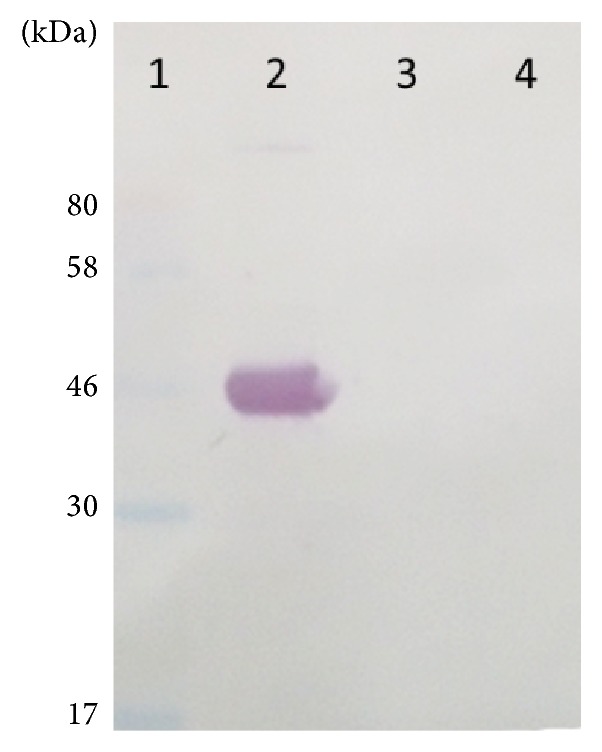
Western blot analysis of ST3Gal-I with 7E5 1C8 3A10 monoclonal antibodies. Lane 1: protein molecular weight marker from NEB; lane 2: purified recombinant ST3Gal-I protein (3 *μ*g); lane 3: negative control 1 (Human Serum); lane 4: negative control 2 (recombinant galectin 3 containing 6x His tag, 31.5 kDa).

The specificity of the monoclonal antibodies was also analyzed by SDS-PAGE followed by western blot. 7E5 1C8 3A10 monoclonal antibodies specifically recognized 44 kDa ST3Gal-I protein. Antibodies did not exhibit any reactivity to normal human serum proteins and His-Tag containing recombinant galectin 3 proteins (Figure 4).

### 3.5. Immunohistochemistry

**Figure 5:**
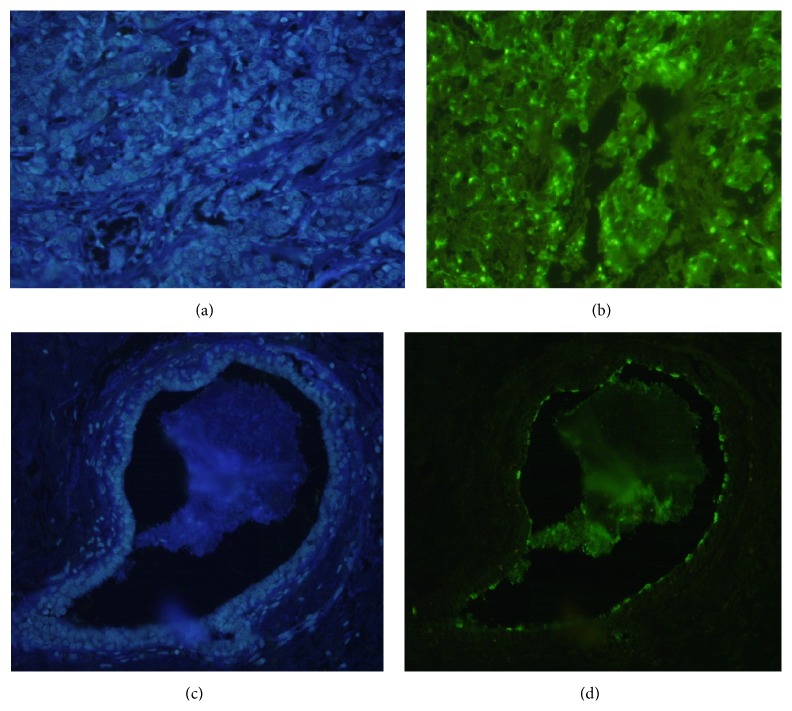
IHC-7E5 1C8 3A10 mAbs against ST3Gal-I (human breast cancer versus human fibroadenoma). (a) DAPI stained human breast cancer tissue section, (b) 7E5 1C8 3A10mAbs stained human breast cancer tissue section, (c) DAPI stained human fibroadenoma tissue section, and (d) 7E5 1C8 3A10mAbs stained human fibroadenoma tissue section.

Strong signal was detected in positive control tissue, that is, clinically diagnosed human breast carcinoma tissues, which was strongly distinguishable despite a small level of expected background staining in negative control (human fibroadenoma tissue, one of the most common benign breast lesions) as shown in Figure 5.

## 4. Discussion

As reported, T antigen is overexpressed in different cancers which include colon, breast, lung, and pancreatic cancer; but it is also expressed in approximately 40% of normal tissues within detectable range [22]. Likewise, the Sialyl-T antigen (ST) is overexpressed in some cancers which include bladder and breast cancer [12, 14, 23] but also detected in normal tissue and leukocytes [24, 25]. ST3Gal-I is responsible for the irreversible conversion of T antigen to Sialyl-T antigen by sialylation. In this study, we have expressed the full length ST3Gal-I protein in* E. coli* and developed a repertoire of monoclonal antibodies against human recombinant ST3Gal-I. R&D System, USA, has expressed the ST3Gal-I protein in NS0-derived, mouse myeloma cells and targeted 57 Arg to 340 Arg amino acid with HA (YPYDVPDYA) tag. The theoretical and reported size (33.4 kDa) are the same which state that there is no or very less glycosylation. Lee et al. expressed the catalytic domain of ST3Gal-I, lacking N terminus 55 amino acid residues, and reported very slight glycosylation [26]. Our preliminary data suggests that mAb (7E5 1C8 3A10) specifically recognizes native ST3Gal-I in cancer tissues and also has shown strong reactivity and specificity with* E. coli* expressed ST3Gal-I by assays such as western blot and ELISA. These data demonstrate the potential of mAbs of 7E5 1C8 3A10 clone to be used as a diagnostic reagent for studying ST3Gal-I expression in tumors and hence expression of T and Sialyl-T antigen. Thus this study may help in glycan research as well as understanding the diagnostic potential of ST3Gal-I in different carcinomas.

## 5. Conclusion

Our strategy was to apply the recombinant* E. coli* expressed ST3Gal-I as an immunogen for the development of highly specific mAbs reactive with cellular ST3Gal-I and recombinant ST3Gal-I. We have successfully purified the recombinant ST3Gal-I from* E. coli* cells and developed monoclonal antibody against ST3Gal-I which specifically detect the human breast cancer tissue with no reactivity with human fibroadenoma tissue. Further, studies are needed to establish ST3Gal-I and its antibody as diagnostic tool for breast cancer diagnosis along with existing biomarkers.

## Abbreviations

LB: Luria broth
IPTG: Isopropyl thio-*β*-D-galactosidase
PAGE: Polyacrylamide gel electrophoresis
C2GnT: Core-2 *β*1 3-N-acetylglucosaminetransferase
C3GnT: Core-3 *β*1,3-N-acetylglucosaminetransferase
CV: Column volumes
IMAC: Immobilized metal affinity chromatography
mAbs: Monoclonal antibodies.
